# An Improved Human-Body-Segmentation Algorithm with Attention-Based Feature Fusion and a Refined Stereo-Matching Scheme Working at the Sub-Pixel Level for the Anthropometric System

**DOI:** 10.3390/e24111647

**Published:** 2022-11-13

**Authors:** Lei Yang, Xiaoyu Guo, Xiaowei Song, Deyuan Lu, Wenjing Cai, Zixiang Xiong

**Affiliations:** 1 School of Electronic and Information, Zhongyuan University of Technology, Zhengzhou 450007, China; 2 Dongjing Avenue Campus, Kaifeng University, Kaifeng 475004, China; 3 Department of Electrical and Computer Engineering, Texas A&M University, College Station, TX 77843, USA

**Keywords:** feature fusion, stereo matching, CBAM, PSPNet, anthropometric

## Abstract

This paper proposes an improved human-body-segmentation algorithm with attention-based feature fusion and a refined corner-based feature-point design with sub-pixel stereo matching for the anthropometric system. In the human-body-segmentation algorithm, four CBAMs are embedded in the four middle convolution layers of the backbone network (ResNet101) of PSPNet to achieve better feature fusion in space and channels, so as to improve accuracy. The common convolution in the residual blocks of ResNet101 is substituted by group convolution to reduce model parameters and computational cost, thereby optimizing efficiency. For the stereo-matching scheme, a corner-based feature point is designed to obtain the feature-point coordinates at sub-pixel level, so that precision is refined. A regional constraint is applied according to the characteristic of the checkerboard corner points, thereby reducing complexity. Experimental results demonstrated that the anthropometric system with the proposed CBAM-based human-body-segmentation algorithm and corner-based stereo-matching scheme can significantly outperform the state-of-the-art system in accuracy. It can also meet the national standards GB/T 2664-2017, GA 258-2009 and GB/T 2665-2017; and the textile industry standards FZ/T 73029-2019, FZ/T 73017-2014, FZ/T 73059-2017 and FZ/T 73022-2019.

## 1. Introduction

Anthropometric data are the basic data of national production and development, which play an important role in costume design, health assessment and industrial design to guarantee a healthy and comfortable user experience [1,2,3,4]. Manual anthropometric measurement mainly depends on the experience of the surveyor, whose accuracy fluctuates with different surveyors and whose efficiency is restricted by the surveyor [5]. With the development of information processing technology, 3D human-body scanners, such as the 3D laser scanner and structured light scanner. have greatly improved the accuracy and efficiency of anthropometric measurement [6,7]. However, such devices typically extract anthropometric data from hundreds of thousands of scanning data, which requires a huge amount of data storage and computation and hinders its widespread application [8,9]. With lower device complexity and less data, the application of optical cameras in anthropometry has attracted more and more attention [10]. Anthropometric devices with optical cameras collect optical images of the human body and perform anthropometry by processing the captured images.

The anthropometric methods in [11,12,13] are based on 2D image processing, in which the intermediate measurement data are obtained by 2D image processing, and the anthropometric data are predicted by substituting the measured data into a mathematical equation for the human body. In references [11], a shape-coding algorithm was adopted to extract feature points from the segmented-human-body contour curve; thus, the anthropometry was completed according to the extracted feature points. In references [12,13], the human body’s circumference was predicted by the constructed regression equation according to the measured width and depth from the front and side images of a subject. Nevertheless, due to the lack of 3D spatial information, the measurement accuracies of these 2D-image-processing-based anthropometric methods are relatively low.

The anthropometric methods in [14,15,16] are based on 3D model reconstruction, in which a 3D human-body model is reconstructed from the point-cloud data obtained by multi-view image processing and the anthropometric data are measured from the reconstructed 3D human-body model. In reference [14], front and rear human-body images were captured by four pairs of stereo cameras, a 3D human-body surface was reconstructed with high-density point clouds obtained by multi-scale matching among multi-view images and the anthropometry was completed on the reconstructed 3D human-body surface. In reference [15], thirty pairs of stereo images were collected by sixty synchronously triggered optical cameras, dense point clouds were extracted by hierarchical stereo matching and a 3D human-body model was reconstructed by multi-view registration and surface meshing, and thus the human-body measurements were completed. In reference [16], ninety human-body images were acquired, sparse human-body point clouds were generated by structure from motion(SFM) and then dense human-body point clouds were recovered by multi-view stereo (MVS), from which the 3D human-body model was reconstructed, and thus the anthropometry was accomplished. Although the measurement accuracies of these 3D-model-reconstruction-based anthropometric methods are high, the reconstruction processes for 3D human-body models from multiple images are extremely complicated and time consuming.

The anthropometric methods in [17,18] make a trade-off between the accuracy and complexity of the aforementioned two types of anthropometric methods with optical cameras. In reference [17], three pairs of synchronously triggered stereo cameras were adopted to collect three pairs of stereo images from the front, side and back of a subject. In reference [18], one pair of stereo cameras and a turntable were used to acquire four pairs of stereo images of a subject from four different views with partially overlapping areas. Both methods made use of the 3D spatial information obtained through stereo matching and coordinate calculation of markers to improve the measurement accuracies, which are greater than those of the 2D-image-processing-based methods in [11,12,13]. Moreover, both methods take advantage of semantic segmentation and girth fitting instead of 3D reconstruction to reduce the measurement complexities; they are less complicated than those of the 3D-model-reconstruction-based methods in [14,15,16]. However, since each marker used for stereo matching usually contains hundreds of pixels, the error of coordinate calculation would be very large if the selected matching point pair were far from the center, which will reduce the anthropometry accuracy. What is more, the accuracy and efficiency of the human-body semantic segmentation can be further optimized.

In this paper, an improved human-body-segmentation algorithm with attention-based feature fusion and a refined corner-based feature-point design with stereo matching at the sub-pixel level are presented for anthropometry. For the human body’s semantic segmentation, the attention mechanism was combined with the segmentation network PSPNet for better space and channel feature fusion. Specifically, four convolutional block attention modules (CBAMs) were embedded in the four middle convolution layers of the backbone network (ResNet101) of PSPNet to improve the segmentation accuracy. What is more, the common convolution in the residual blocks of ResNet101 was replaced with group convolution to optimize the segmentation efficiency. For the stereo matching, the checkerboard corner was designed to replace the color marker; thus, the Shi–Tomasi corner detection-based stereo matching with regional constraint is proposed to replace the SURF-based stereo matching with a cluster constraint. The matching precision is refined to the sub-pixel level by the checkerboard corner design and the corresponding corner detection algorithm, and the matching complexity is reduced by the regional constraint of the checkerboard corner. The proposed algorithm and design can significantly improve the accuracy of the anthropometric system in [17,18].

The rest of the paper is organized as follows. In Section 2, we review some related works on segmentation and attention mechanisms. In Section 3, we propose an improved human-body-segmentation algorithm with attention-based feature fusion and a refined corner-based feature-point design with sub-pixel stereo matching. In Section 4, we report the experimental results. In Section 5, we draw conclusions.

## 2. Related Works

Semantic segmentation classifies each pixel in the image and extracts the region of interest (ROI) from the background [19,20], which is very beneficial for efficient stereo matching [21,22,23] in anthropometry if the human-body segmentation is accurate. The fully convolutional network (FCN) [24] is the foundation of semantic segmentation. It successfully extends the classification from the image level to the pixel level by replacing the full connection (FC) layer with the convolution layer. However, a FCN does not effectively consider the context information of the image, and some spatial information at the pixel level is lost [25]. Therefore, many improved semantic segmentation methods have emerged since then, which can be divided into three categories: FCN-based methods [25], encoder-decoder-based methods [26] and feature-fusion-based methods [27]. For the FCN-based methods, such as DeepLab [25], DeepLabv2 [28] and DeepLabv3 [29], the sensitivity field of the filter is enhanced by atrous convolution, the multi-scale representation of the image is achieved and the spatial accuracy of the segmentation result is improved. However, the segmentation speed is slow and the segmentation for small scale objects is not good. For the encoder–decoder-based methods, such as SegNet [26], Unet [30] and DeconvNet [31], the pixel position information of the image is restored by deconvolution and up-pooling or bilinear interpolation, so as to better reflect the object details and avoid the resolution reduction of the feature map caused by the pooling operation. Nevertheless, they also fail to take full advantage of the context information of the image. For the feature-fusion-based methods, such as PSPNet [27], RefineNet [32] and ICNet [33], feature information fusion of different scales and from different positions is achieved by a pyramid pooling module (PPM), multi-scale convolution module and cascade module; thus, the segmentation result is refined. Among them, PSPNet is the one with the smallest network capacity and fastest processing speed, which considers both global semantic information and local detailed information, fuses the feature information and improves the segmentation accuracy. Hence, PSPNet is applied to segment human-body regions, which confines stereo matching to smaller areas and improves the anthropometric efficiency.

However, in the feature-extraction stage of PSPNet, all features are given the same weight, resulting in excessive allocation of computing resources to invalid feature extraction. If more computing resources can be allocated to the features of attention, the segmentation accuracy of PSPNet can be further improved. An attention mechanism helps to allocate more available computing resources to the target region to be segmented, so as to achieve better space and channel feature fusion. Some attention models have been used to guide the deep-learning-based human-body segmentation [34]. An attention-guided progressive partition network (APPNet) with a global attention module (GAM) was proposed in [35]. Features are given different weights in the spatial dimension according to the global attention, which focuses the significance detection on the human-body segmentation and improves the feature learning ability of the model. A trilateral awareness operation (TAO) is provided in [36]. The spatial attention and channel attention are combined with the dilation convolution, which enhances the CNN’s perceptive ability of multi-scale feature information and achieves fine-grained human-body segmentation. A mutual attention structure is presented in [37]. The feature map is recalibrated in the spatial and channel dimensions, which increases the spatial perception and the cross-channel context perception of the human-body-segmentation. Given these attention-based methods, the PSPNet selected can be further improved by combining it with the attention module to achieve better spatial and channel feature fusion, and thus improve the human-body segmentation precision.

The attention modules can be divided into three types: channel attention module [38], space attention module [39] and mixed attention module [40]. The channel attention module concentrates on optimizing cross-channel context information and reinforcing semantic information, and the spatial attention module focuses on optimizing location features and enhancing spatial perception. The mixed attention module considers both and fuses important feature information in both channel and space. The typical mixed attention module is CBAM [41]. For CBAM, features are extracted in both channel and spatial dimensions, and the attention map is multiplied by the input feature map for adaptive feature refinement. The representational ability of the network can be improved from both channel and spatial dimensions, thereby further improving the performance of semantic segmentation.

## 3. The Proposed Method

An improved human-body-segmentation algorithm with attention-based feature fusion and a refined corner-based feature-point design with sub-pixel stereo matching for the stereovision-based anthropometric system are proposed in this paper. The proposed human-body-segmentation algorithm aims to improve the segmentation accuracy and reduce the number of parameters of the model. The proposed feature-point design aims to improve the stereo-matching accuracy and reduce the matching complexity.

**Figure 1:**
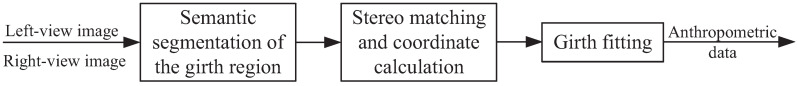
Flowchart of the stereovision-based anthropometric system.

The process of the stereovision-based anthropometry can be divided into three steps: semantic segmentation of the girth region; stereo matching and coordinate calculation; and girth fitting [17,18]. The flowchart is shown in Figure 1.

In the semantic segmentation process, the girth region is segmented to confine the subsequent stereo matching to a smaller area, so as to increase the matching accuracy and efficiency. The higher the segmentation precision, the better the matching effect. Therefore, the semantic segmentation network PSPNet can be further improved to enhance the performance. In this paper, the feature extraction of human-body contour and semantic information is optimized by CBAM. Four CBAMs were added to the middle convolution layers of ResNet101 to refine the features of human-body segmentation. Moreover, in the residual blocks of ResNet101, the group convolution was chosen to replace the common convolution, so as to reduce the computational overhead.

**Figure 2:**
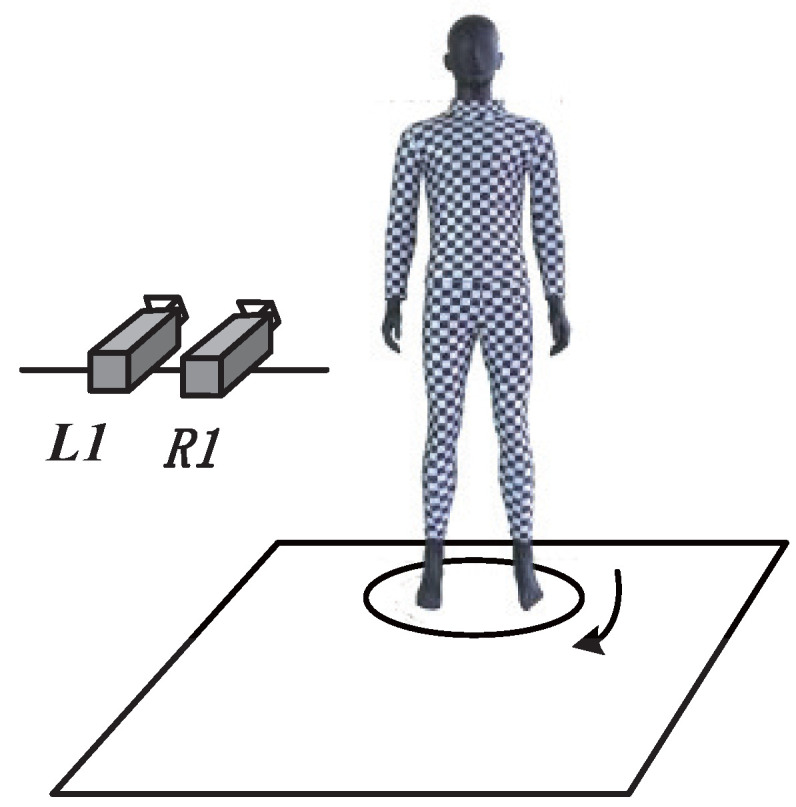
Binocular stereovision-based anthropometric system with checkerboard corner design.

In the stereo matching and coordinate calculation process, the matching point pairs are obtained by SURF matching based on color and spatial clustering of the markers. The matching point pair closest to the marker center is selected from the obtained multiple matching point pairs within the marker range as the stereo-matching result of that marker, so as to perform coordinate calculation. However, in obtaining the matching point pairs, there are usually hundreds of pixels with similar characteristic in the range of a same marker, so the matching error may be large, and it is difficult to ensure that the selected matching point pair is close enough to the marker center. As a result, the accuracy of anthropometry is not high enough. In this paper, as shown in Figure 2, a checkerboard corner design is proposed to replace the color marker design, in which the subject wears tights with a black and white checkerboard pattern for measurement, with 2.5 cm spacing between adjacent checkerboards. Shi–Tomasi corner detection is used to get the feature-point set in the segmented human-body region, and regional constraining is performed on the obtained feature-point set according to the location information of two preset color markers and the characteristic of the checkerboard, so as to acquire the matching point pair of the same feature point in the left and right images. Hence, the refined stereo matching at sub-pixel level is achieved.

In the girth fitting process, the feature points rotating along with the turntable are reversely rotated to their initial positions, then polynomical with intermediate variable curve fitting (PIVCF) is used to achieve anthropometry.

### 3.1. A Human-Body-Segmentation Algorithm Based on a CBAM Attention Mechanism

To increase the segmentation accuracy, it is necessary to focus on the human-body region to be segmented and suppress useless information as much as possible. Due to the fixed distance of the camera and the predetermined posture of the subject, the same category of region to be segmented is located at almost the same position in the image. Therefore, the semantic segmentation network should have strong spatial perception. What is more, different categories of regions to be segmented are similar in size and prone to mis-segmentation. Thus, the network should have strong semantic information perception and cross-channel context information fusion ability [42]. The CBAM attention mechanism can focus on the space and channel information at the same time; realize the feature fusion of space and channel; enhance the perception of spatial and semantic information of the network; and improve the segmentation performance. Hence, CBAM was selected in this paper to further enhance the segmentation performance of PSPNet.

**Figure 3:**
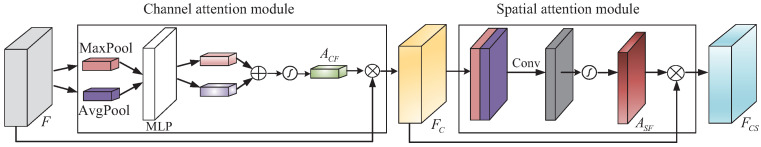
CBAM schematic diagram.

In CBAM [41], as shown in Figure 3, the channel attention module performs maximum pooling and average pooling on the input feature map *F* to obtain two 1D vectors which represent the channel information of *F* in the local and global features, respectively, and aggregate the spatial information as well. Then, the two 1D vectors are input into a multi-layer perception (MLP) for interaction, and the two perceived 1D vectors are added element by element. Finally, a 1D channel attention map $A_{CF}$ is generated through the sigmoid activation function and is multiplied with the input feature map *F* to obtain the channel refined feature map $F_{C}$.

(1) $$F_{C}=F⊗A_{CF}=F⊗Sig\left(\mathrm{MLP}\left(\mathrm{AvgPool}\left(F\right)\right)⊕\mathrm{MLP}\left(\mathrm{MaxPool}\left(F\right)\right)\right)$$

wherein ⊗ denotes element-wise multiplication, Sig denotes the sigmoid activation function and ⊕ denotes element-wise addition.

The spatial attention module performs maximum pooling and average pooling along the channel axis on the channel-refined feature map $F_{C}$ to obtain two 2D vectors which represent the spatial information of $F_{C}$ in terms of local and global features. Then, the two 2D vectors are cascaded and convolved. Finally, a 2D spatial attention map $A_{SF}$ is generated through the sigmoid activation function and is multiplied with $F_{C}$ to obtain the space- and channel-refined feature map $F_{CS}$.

(2) $$F_{CS}=F_{C}⊗A_{SF}=F_{C}⊗Sig\left(f^{7\times 7}\left(\left[\mathrm{AvgPool}\left(F_{C}\right);\mathrm{MaxPool}\left(F_{C}\right)\right]\right)\right)$$

wherein ⊗ denotes element-wise multiplication, Sig denotes the sigmoid activation function, $f^{7\times 7}$ denotes the convolution layer with a 7×7 convolution kernel and [;] denotes cascade.

**Figure 4:**
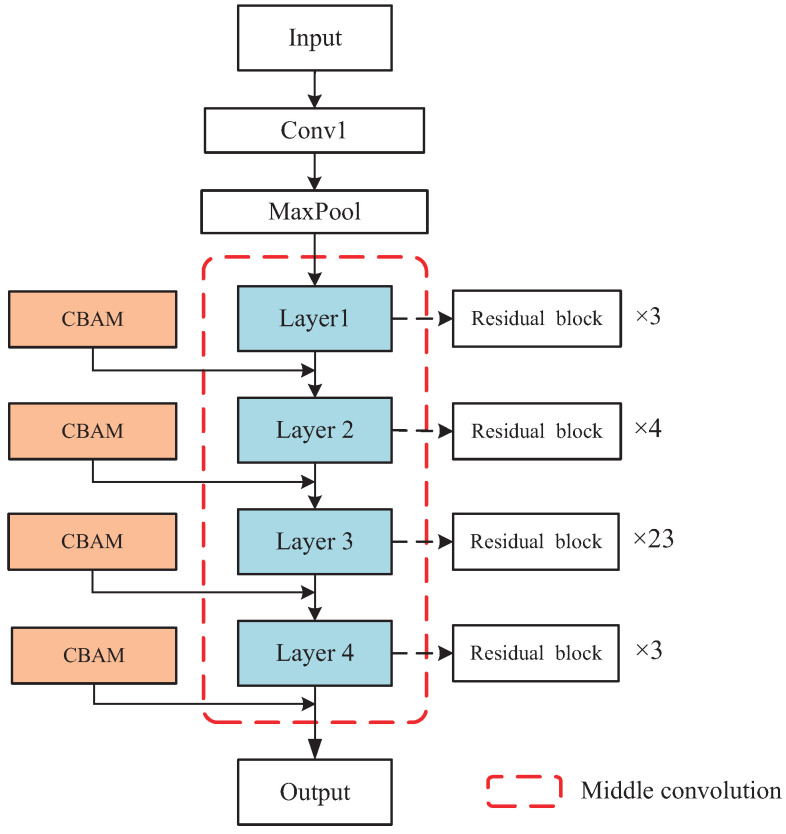
Backbone network structural diagram of CBAM-PSPNet.

**Figure 5:**
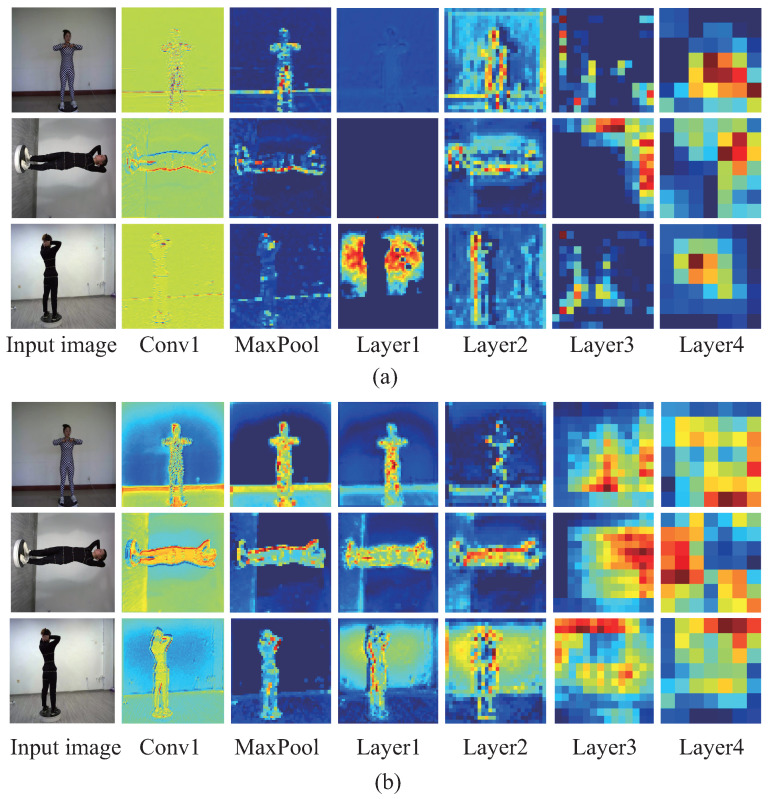
Visualization comparison of feature maps in backbone networks between PSPNet and CBAM-PSPNet. (**a**) PSPNet. (**b**) CBAM-PSPNet.

ResNet101 consists of three parts: the input part, the middle convolution part (layer 1–4) and the output part. The middle convolution part is constructed from residual blocks, among which there are 3 residual blocks in layer1, 4 residual blocks in layer2, 23 residual blocks in layer3, and 3 residual blocks in layer4. Figure 4 shows the specific embedded positions of CBAMs in the middle convolution layers of the backbone network (ResNet101) of PSPNet. A CBAM is embedded in the output of each of the four layers. Figure 5 shows the visualization comparison of feature maps between the backbone network of PSPNet and that of CBAM-PSPNet. The visualization of six feature maps in the feature extraction stage is compared, corresponding to the outputs of Conv1, MaxPool, Layer1, Layer2, Layer3 and Layer4 in Figure 4. According to the visual effect, there is a significant improvement in the extraction of low-level edge information, i.e., human-contour information for CBAM-PSPNet in the feature extraction stage of Conv1, MaxPool, Layer1 and Layer2. Moreover, there is a moderate improvement in the extraction of high-level schematic information, i.e., richer schematic information for CBAM-PSPNet in the feature extraction stage of Layer3 and Layer4. Therefore, the improved CBAM-PSPNet can achieve adaptive feature refinement of the input feature map, along with better spatial perception and cross-channel context information fusion.

Furthermore, to reduce the computational cost of the network, the common convolution in the residual blocks of the backbone network is replaced by the group convolution according to its characteristic that the number of parameters in the model reduces with an increase in the number of groups. Assume that the size of an input feature is $H_{in}\times W_{in}\times D_{in}$ and the size of an output feature is $H_{\mathrm{out}\;}\times W_{\mathrm{out}\;}\times D_{\mathrm{out}\;}$. For common convolution, there are $D_{\mathrm{out}\;}$ convolution kernels of size $h\times w\times D_{in}$, and the parameter number $P_{1}$ can be calculated by Equation (3).

(3) $$P_{1}=h\times w\times D_{in}\times D_{out}$$

For group convolution, assuming *g* groups, there are $\frac{D_{\mathrm{out}\;}}{g}$ convolution kernels of size $h\times w\times \left(\frac{D_{in}}{g}\right)$ in each group, and the parameter number $P_{2}$ can be calculated by Equation (4) [43].

(4) $$P_{2}=h\times w\times \left(\frac{D_{in}}{g}\right)\times \left(\frac{D_{\mathrm{out}\;}}{g}\right)\times g=\frac{h\times w\times D_{in\;}\times D_{\mathrm{out}\;}}{g}=\frac{P_{1}}{g}$$

**Figure 6:**
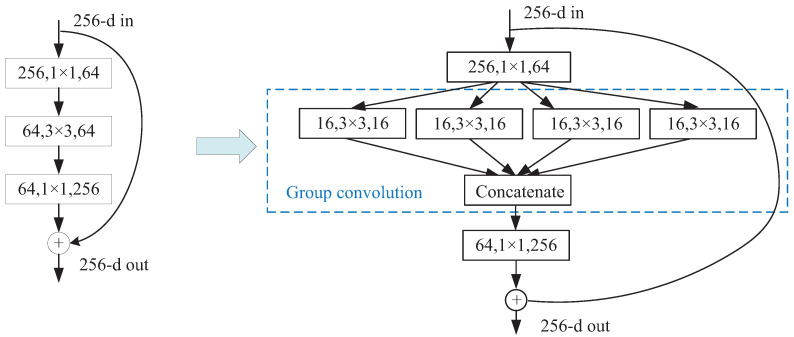
Structural chart of the residual block from the common convolution to the group convolution.

As shown in Equation (4), the parameter number of the group convolution is $\frac{1}{g}$ of the common convolution, which reduces the number of parameters in the model and improves the segmentation efficiency. Figure 6 is the structural chart of the residual block from the common convolution to the group convolution. For a 256-d input feature map, the output is obtained by processing the input through two branches, a linear branch and a shortcut branch. Sixty-four common convolution kernels of size 3×3×64 in the second layer of the residual block are replaced by four groups of convolution kernels; each group has 16 convolution kernels of size 3×3×16. Then, the four outputs of each group are concatenated. The parameter number of the second layer of the residual block is reduced from $P_{1}=3\times 3\times 64\times 64$ to $P_{2}=3\times 3\times \left(\frac{64}{4}\right)\times \left(\frac{64}{4}\right)\times 4=3\times 3\times 16\times 16\times 4=\frac{P_{1}}{4}$.

**Figure 7:**
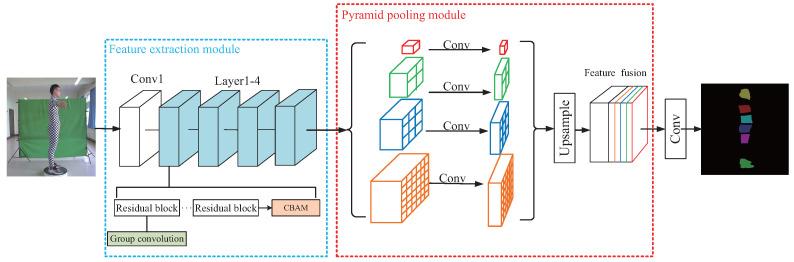
Schematic diagram of CBAM-PSPNet.

Figure 7 shows the schematic diagram of CBAM-PSPNet. Firstly, a feature extraction module extracts the contour features, position features, etc., of the human-body parts from the input image, and generates a feature map containing both channel and spatial attention, which will improve the segmentation accuracy. The feature extraction module is improved by embedding a CBAM module at the end of each layer (1–4) of the backbone network and substituting group convolutions in the second layer of each of the residual blocks in each layer. Then, the pyramid pooling module extracts the context information of the generated feature map. The pyramid pooling kernels have four levels, that is, 1×1, 2×2, 3×3 and 6×6, in which the global and local features of different scales are extracted. Next, the features extracted in the four levels and the input features are fused to form a composite feature map which contains both global and local context information. Finally, the human-body segmentation is achieved by the convolution of the input feature map with the composite feature map.

Table 1 shows a comparison of the number of parameters and computational cost between the improved ResNet101 and the original ResNet101. For the input feature map of size 224×224, the number of parameters in ResNet101 is 42.50 million, and the computational cost is 7.84 billion FLOPs. The number of parameters in the improved ResNet101 is 32.52 million, a reduction of 23.5%; and the computational cost is 5.94 billion FLOPs, a reduction of 24.2%. The reductions in the number of parameters and computational cost are mainly attributed to the group convolution substitution, and the experimental data are consistent with the theoretical analysis mentioned above.

To verify the performance of CBAM-PSPNet, 15,795 human-body images were selected as the training set and 4513 human-body images were selected as the test set. Table 2 shows the performance comparison between CBAM-PSPNet and PSPNet. The pixel accuracy (PA) of PSPNet was 98.36%, the mean pixel accuracy (MPA) was 88.25% and the mean intersection over union (MIOU) was 82.30%. The PA of CBAM-PSPNet was 98.39%, an increase of 0.03%; the MPA was 92.28%, an increase of 4.03%; and the MIOU was 83.11%, an increase of 0.81%. The increases in accuracy can be mainly attributed to the embedding of CBAMs, which helps to generate feature maps that simultaneously fuse channel attention and spatial attention, so as to improve the segmentation accuracy.

### 3.2. Refined Corner-Based Stereo-Matching Scheme Working at the Sub-Pixel Level

**Figure 8:**
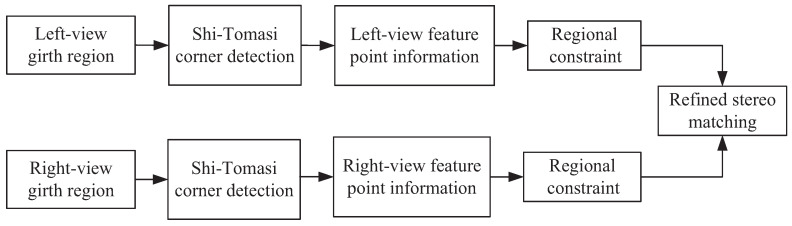
Schematic diagram of the refined stereo matching at sub-pixel level based on the corner design.

The feature-point design directly affects the matching accuracy, and the matching accuracy directly determines the anthropometry accuracy. Figure 2 has shown the checkerboard corner design proposed in this paper for optimizing anthropometry accuracy. Figure 8 shows the schematic diagram of the refined stereo-matching scheme that works at the sub-pixel level based on the corner design in Figure 2. In the anthropometry of this paper, firstly, the left-view and right-view girth regions of human body were segmented by CBAM-PSPNet. Next, the Shi–Tomasi corner detection algorithm was used to extract the feature-point information at the sub-pixel level in the girth region. Then, a regional constraint was applied to the extracted feature-point set of corners according to the characteristics of the color markers and the checkerboard. Finally, refined stereo matching on a baseline in the region was realized according to the characteristics of corner coordinates, and refined stereo matching on multi-lines in the region was achieved according to the characteristics of the checkerboard, so as to further improve the accuracy of human-body girth measurement.

**Figure 9:**
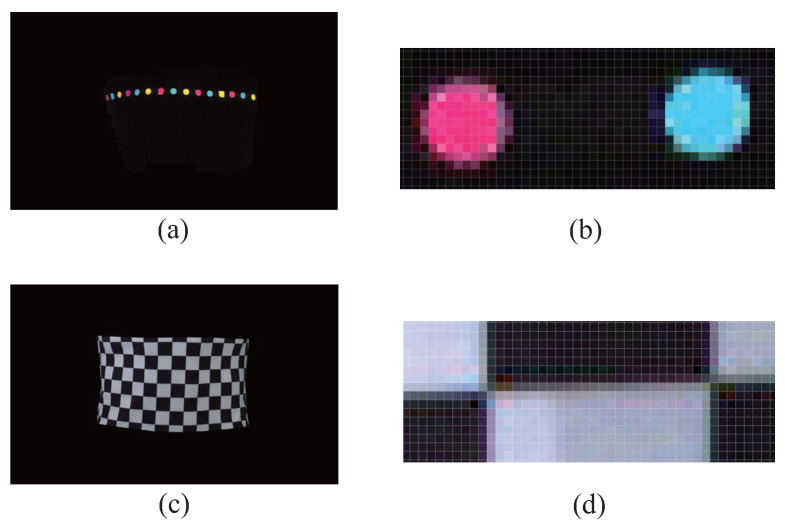
Comparison of pixel numbers. (**a**) Segmented image in [17,18]. (**b**) Partial enlarged view of (**a**), (**c**) Segmented image in this paper. (**d**) Partial enlarged view of (**c**).

In the anthropometric system in reference [17,18], color markers are used for stereo matching, and the matching point pair closest to the center of the marker is reserved for spatial coordinate calculation. In the anthropometric system in this paper, corners are used for stereo matching. Figure 9 shows the pixel number comparison between the color markers and the corners in the same shooting conditions and with the same magnification. Figure 9a is the segmented image of human-body parts in reference [17,18], and Figure 9b is a partial, enlarged view of the color markers. Figure 9c is the segmented image of the same part in this paper, and Figure 9d shows the partial, enlarged view of the corners. Since the feature-point matching is carried out within the range of the color marker or the corner, the sizes of the color marker and the corner determine the search range for feature-point matching. As shown in Figure 9b,d, a color marker contains hundreds of pixels, whereas a corner only includes four pixels. Therefore, the corner design proposed in this paper can greatly reduce the search range of feature-point matching and achieve fast and accurate matching.

**Figure 10:**
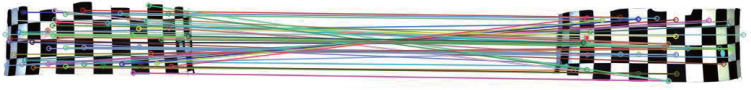
SURF matching result for the segmented corner-based images.

Figure 10 shows the result of SURF matching [44] on the corner-based segmented images. Due to the high similarity between the detected feature points on the checkboard, there must be a lot of mismatches in SURF matching. For example, in Figure 10, a total of 38 pairs of matching points exist, among which 29 pairs are mismatched and only 9 pairs are matched. This mismatching rate is 76.3%, which is too high to eliminate the mismatching points. Moreover, the SURF-detected feature points are mostly not the checkboard corners, which is not beneficial for accurate girth measurement. Therefore, SURF matching is no longer suitable for feature-point matching in this paper. It is necessary to find a more effective matching method for the checkerboard corners. As shown in Figure 8, a refined stereo-matching method that works at a sub-pixel level based on the characteristics of corners is proposed in this paper.

**Figure 11:**
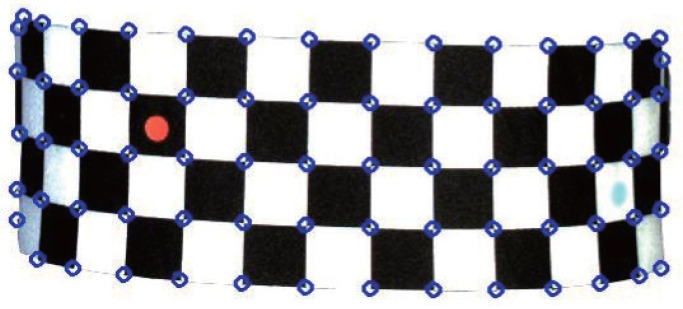
Shi–Tomasi detection result.

For the left-view and right-view human-body regions segmented by the CBAM-PSPNet human-body-segmentation algorithm, the checkerboard corners need to be detected as accurately as possible. The commonly used corner feature detection methods include Harris and Shi–Tomasi’s methods [45]. The Shi–Tomasi detector [46] has a similar gradient-based mathematical foundation to the Harris detector [47], but with higher accuracy, faster speed and fewer parameters. Therefore, the Shi–Tomasi corner detection algorithm was chosen to accurately locate the corners according to the characteristic of gray value variation in the corner neighborhood. Figure 11 shows the detection result by the Shi–Tomasi corner detection algorithm. The hollow blue dots in Figure 11 represent the positions of the detected corners. Not only could all corners be detected, but the detection accuracy reached the sub-pixel level, which can greatly improve the accuracy of the subsequent stereo matching.

**Figure 12:**
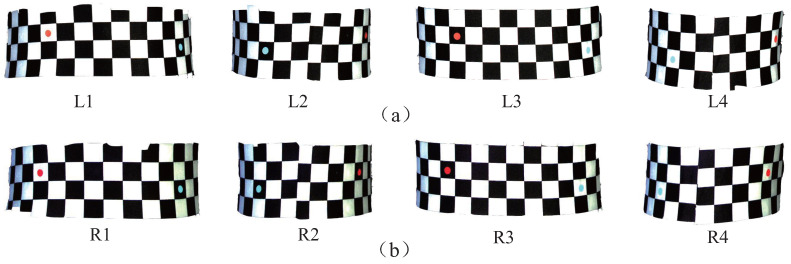
Examples of the preset color markers in the waist region. (**a**) Left view. (**b**) Right view.

Next, according to the characteristics of checkerboard corners, the complexity of stereo matching is reduced by regional constraint. A few color markers were preset at the girth measurement region to assist the regional constraint. Figure 12 shows examples of the preset color markers in the waist region. L1, L2, L3 and L4 are the left-view images of the waist region captured from four different rotation angles of the turntable, respectively; and R1, R2, R3 and R4 are the corresponding right-view images. In each segmented image, a red marker and a cyan marker are shown. A total of four markers were preset to ensure that each image would contain one red marker and one cyan marker. The horizontal distances were 8, 7, 8 and 7 checkerboard intervals from L1(R1) to L4(R4), and the vertical distances were −1, +1, −1 and +1 checkerboard intervals, so that the rectangular area determined by the two markers would contain the same baseline for girth measurement.

In the segmented image, there are four colors, namely, red, cyan, black and white. All pixels in the segmented image constitute a dataset $Z=\left\{z_{i},i=1,2,\cdots ,N\right\}$, wherein $z_{i}$ represents a pixel and *N* is the total number of pixels in the segmented image. Each pixel $z_{i}$ can be expressed as $z_{i}\left(H_{i},S_{i},V_{i}\right)$ in the HSV color space and $z_{i}\left(x_{i},y_{i}\right)$ in the 2D coordinates of the segmented image. Table 3 shows the HSV ranges corresponding to the four colors. If $V_{i}$ is greater than 46, $S_{i}$ is greater than 43 and $H_{i}$ is greater than 0 but less than 10 or $H_{i}$ is greater than 156 and less than 180, the color of $z_{i}$ is red. If $V_{i}$ is greater than 46, $S_{i}$ is greater than 43 and $H_{i}$ is greater than 78 but less than 99, the color of $z_{i}$ is cyan. If $V_{i}$ is greater than 221 and $S_{i}$ is less than 30, the color of $z_{i}$ is white. If $V_{i}$ is less than 46, the color of $z_{i}$ is black. Thus, the pixel set of the red marker in the segmented image is extracted from Z as a smaller dataset $M_{R}=\left\{z_{R}\in Z|V_{R}>46\&S_{R}>43\&\left(0<H_{R}<10∥156<H_{R}<180\right)\right\}$, and the pixel set of the cyan marker in the segmented image is also extracted from Z as another smaller dataset $M_{C}=\left\{z_{C}\in Z|V_{C}>46\&S_{C}>43\&\left(78<H_{C}<99\right)\right\}$, wherein the subscripts *R* and *C* stand for red and cyan, respectively. Taking the waist segmentation images L1 and R1 as examples, a total of four pixel sets of the red and cyan markers for the left and right views are obtained, denoted as $M_{l_{-}R}$, $M_{r_{-}R}$, $M_{l_{-}C}$ and $M_{r_{-}C}$, wherein the subscript *l* and *r* represent the left view and right view, respectively, and the subscript *R* and *C* denote red and cyan, respectively.

(5) $$\begin{array}{cc} \; & M_{l_{-}R}=\left\{z_{l_{-}R_{-}i}\left(x_{l_{-}R_{-}i},y_{l_{-}R_{-}i}\right),i=1,2,\ldots ,N_{l_{-}R}\right\}\\ \; & M_{r_{-}R}=\left\{z_{r_{-}R_{-}i}\left(x_{r_{-}R_{-}i},y_{r_{-}R_{-}i}\right),i=1,2,\ldots ,N_{r_{-}R}\right\}\\ \; & M_{l_{-}C}=\left\{z_{l_{-}C_{-}i}\left(x_{l_{-}C_{-}i},y_{l_{-}C_{-}i}\right),i=1,2,\ldots ,N_{l_{-}C}\right\}\\ \; & M_{r_{-}C}=\left\{z_{r_{-}C_{-}i}\left(x_{r_{-}C_{-}i},y_{r_{-}C_{-}i}\right),i=1,2,\ldots ,N_{r_{-}C}\right\} \end{array}$$

wherein $z_{l_{-}R_{-}i}$, $z_{r_{-}R_{-}i}$, $z_{l_{-}C_{-}i}$ and $z_{r_{-}C_{-}i}$ represent the pixels in $M_{l_{-}R}$, $M_{r_{-}R}$, $M_{l_{-}C}$ and $M_{r_{-}C}$, respectively; $N_{l_{-}R}$, $N_{r_{-}R}$, $N_{l_{-}C}$ and $N_{r_{-}C}$ are the total numbers of pixels in $M_{l_{-}R}$, $M_{r_{-}R}$, $M_{l_{-}C}$ and $M_{r_{-}C}$, respectively. Specifically, $x_{l_{-}R_{-}i}$ and $y_{l_{-}R_{-}i}$ are the 2D coordinates of the pixel $z_{l_{-}R_{-}i}$ in the pixel set $M_{l_{-}R}$, $x_{r_{-}R_{-}i}$, $y_{r_{-}R_{-}i}$ are the 2D coordinates of the pixel $z_{r_{-}R_{-}i}$ in the pixel set $M_{r_{-}R}$, $x_{l_{-}C_{-}i}$, $y_{l_{-}C_{-}i}$ are the 2D coordinates of the pixel $z_{l_{-}C_{-}i}$ in the pixel set $M_{l_{-}C}$, $x_{r_{-}C_{-}i}$ and $y_{r_{-}C_{-}i}$ are the 2D coordinates of the pixel $z_{r_{-}C_{-}i}$ in the pixel set $M_{r_{-}C}$. Thus, the central points of the red and cyan markers in L1 and R1, that is, ${\bar{z}}_{l_{-}R}\left({\bar{x}}_{l_{-}R},{\bar{y}}_{l_{-}R}\right)$, ${\bar{z}}_{r_{-}R}\left({\bar{x}}_{r_{-}R},{\bar{y}}_{r_{-}R}\right)$, ${\bar{z}}_{l_{-}C}\left({\bar{x}}_{l_{-}C},{\bar{y}}_{l_{-}C}\right)$ and ${\bar{z}}_{r_{-}C}\left({\bar{x}}_{r_{-}C},{\bar{y}}_{r_{-}C}\right)$, are calculated by averaging all the pixels in the respective pixel sets $M_{l_{-}R}$, $M_{r_{-}R}$, $M_{l_{-}C}$ and $M_{r_{-}C}$, as shown in Equations (6):

(6) $$\begin{array}{cc} \; & {\bar{x}}_{l_{-}R}=\frac{1}{N_{l_{-}R}}\sum_{i=1}^{N_{l_{-}R}}x_{l_{-}R_{-}i},\;{\bar{y}}_{l_{-}R}=\frac{1}{N_{l_{-}R}}\sum_{i=1}^{N_{l_{-}R}}y_{l_{-}R_{-}i}\\ \; & {\bar{x}}_{r_{-}R}=\frac{1}{N_{r_{-}R}}\sum_{i=1}^{N_{r_{-}R}}x_{r_{-}R_{-}i},\;{\bar{y}}_{r_{-}R}=\frac{1}{N_{r_{-}R}}\sum_{i=1}^{N_{r_{-}R}}y_{r_{-}R_{-}i}\\ \; & {\bar{x}}_{l_{-}C}=\frac{1}{N_{l_{-}C}}\sum_{i=1}^{N_{l_{-}C}}x_{l_{-}C_{-}i},\;{\bar{y}}_{l_{-}C}=\frac{1}{N_{l_{-}C}}\sum_{i=1}^{N_{l_{-}C}}y_{l_{-}C_{-}i}\\ \; & {\bar{x}}_{r_{-}C}=\frac{1}{N_{r_{-}C}}\sum_{i=1}^{N_{r_{-}C}}x_{r_{-}C_{-}i},\;{\bar{y}}_{r_{-}C}=\frac{1}{N_{r_{-}C}}\sum_{i=1}^{N_{r_{-}C}}y_{r_{-}C_{-}i} \end{array}$$

wherein ${\bar{x}}_{l_{-}R}$ and ${\bar{y}}_{l_{-}R}$ are the 2D coordinates of the central point ${\bar{z}}_{l_{-}R}$ for the pixel set $M_{l_{-}R}$, ${\bar{x}}_{r_{-}R}$, ${\bar{y}}_{r_{-}R}$ are the 2D coordinates of the central point ${\bar{z}}_{r_{-}R}$ for the pixel set $M_{r_{-}R}$, ${\bar{x}}_{l_{-}C}$, ${\bar{y}}_{l_{-}C}$ are the 2D coordinates of the central point ${\bar{z}}_{l_{-}C}$ for the pixel set $M_{l_{-}C}$, ${\bar{x}}_{r_{-}C}$ and ${\bar{y}}_{r_{-}C}$ are the 2D coordinates of the central point ${\bar{z}}_{r_{-}C}$ for the pixel set $M_{r_{-}C}$.

By the Shi–Tomasi corner detection algorithm, the corner sets in the segmented images L1 and R1 are extracted at the sub-pixel level, denoted as $S_{l}=\left\{z_{l\_\mathrm{corner}\_i}\left(x_{l\_corner\_i},y_{l\_corner\_i}\right),i=1,2,\ldots ,N_{l\_corner}\right\}$ and $S_{r}=\left\{z_{r\_\mathrm{corner}\_i}\left(x_{r\_corner\_i},y_{r\_corner\_i}\right),i=1,2,\ldots ,N_{r\_corner}\right\}$, wherein $z_{l\_\mathrm{corner}\_i}$ and $z_{r\_\mathrm{corner}\_i}$ represent the extracted corners from L1 and R1; $N_{l\_corner}$ and $N_{r\_corner}$ are the total numbers of corners in L1 and R1; $x_{l\_corner\_i}$ and $y_{l\_corner\_i}$ are the 2D coordinates of the corner $z_{l\_\mathrm{corner}\_i}$ in the corner set $S_{l}$, $x_{r\_corner\_i}$; and $y_{r\_corner\_i}$ are the 2D coordinates of the corner $z_{r\_\mathrm{corner}\_i}$ in the corner set $S_{r}$.

**Figure 13:**
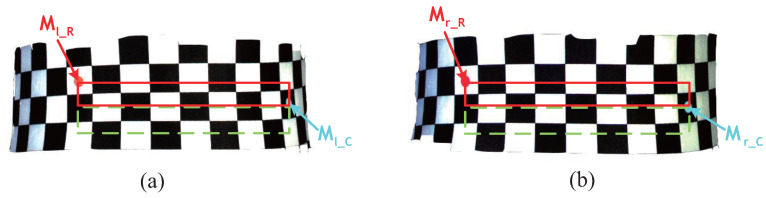
Schematic diagram of corner stereo matching by the regional constraint of markers. (**a**) Left view. (**b**) Right view.

A rectangular region can be determined according to the central point coordinates of the red and cyan markers calculated above. Figure 13 shows an example of the corner matching by the regional constraining of markers. In L1, with the central points of markers ${\bar{z}}_{l_{-}R}\left({\bar{x}}_{l_{-}R},{\bar{y}}_{l_{-}R}\right)$ and ${\bar{z}}_{l_{-}C}\left({\bar{x}}_{l_{-}C},{\bar{y}}_{l_{-}C}\right)$ as the regional constraint, a smaller corner set $S_{l_{-}RC}$ in the rectangular region defined by the red and cyan markers can be obtained, as expressed in Equation (7). In R1, with the central points of markers ${\bar{z}}_{r_{-}R}\left({\bar{x}}_{r_{-}R},{\bar{y}}_{r_{-}R}\right)$ and ${\bar{z}}_{r_{-}C}\left({\bar{x}}_{r_{-}C},{\bar{y}}_{r_{-}C}\right)$ as the regional constraint, another smaller corner set $S_{r_{-}RC}$ in the rectangular region defined by the red and cyan markers can be obtained in the same way, as expressed in Equation (8).

(7) $$\begin{array}{c} S_{l_{-}\mathrm{RC}}=\{z_{l\_\mathrm{corner}\_\mathrm{RC}}\in S_{l}|\left(\min\left\{{\bar{x}}_{l_{-}R},{\bar{x}}_{l_{-}C}\right\}<x_{l\_corner\_RC}<\max\left\{{\bar{x}}_{l_{-}R},{\bar{x}}_{l_{-}C}\right\}\right)\&\\ \;\;\;\;\;\;\;\;\;\;\;\;\;\;\;\;\;\;\;\;\;\;\left(\min\left\{{\bar{y}}_{l_{-}R},{\bar{y}}_{l_{-}C}\right\}<y_{l\_corner\_RC}<\max\left\{{\bar{y}}_{l_{-}R},{\bar{y}}_{l_{-}C}\right\}\right)\} \end{array}$$

(8) $$\begin{array}{c} S_{r_{-}\mathrm{RC}}=\{z_{r\_\mathrm{corner}\_\mathrm{RC}}\in S_{r}|\left(\min\left\{{\bar{x}}_{r_{-}R},{\bar{x}}_{r_{-}C}\right\}<x_{r\_corner\_RC}<\max\left\{{\bar{x}}_{r_{-}R},{\bar{x}}_{r_{-}C}\right\}\right)\&\\ \;\;\;\;\;\;\;\;\;\;\;\;\;\;\;\;\;\;\;\;\;\;\left(\min\left\{{\bar{y}}_{r_{-}R},{\bar{y}}_{r_{-}C}\right\}<y_{r\_corner\_RC}<\max\left\{{\bar{y}}_{r_{-}R},{\bar{y}}_{r_{-}C}\right\}\right)\} \end{array}$$

wherein $z_{l\_\mathrm{corner}\_\mathrm{RC}}$ and $z_{r\_\mathrm{corner}\_\mathrm{RC}}$ represent the corners in the rectangular region of L1 and R1, respectively; $x_{l\_corner\_RC}$ and $y_{l\_corner\_RC}$ are the 2D coordinates of $z_{l\_\mathrm{corner}\_\mathrm{RC}}$; ${\bar{x}}_{l_{-}R}$ and ${\bar{y}}_{l_{-}R}$ are the 2D coordinates of the central point for the red marker in L1; ${\bar{x}}_{l_{-}C}$ and ${\bar{y}}_{l_{-}C}$ are the 2D coordinates of the central point for the cyan marker in L1; $x_{r\_corner\_RC}$ and $y_{r\_corner\_RC}$ are the 2D coordinates of $z_{r\_\mathrm{corner}\_\mathrm{RC}}$; ${\bar{x}}_{r_{-}R}$ and ${\bar{y}}_{r_{-}R}$ are the 2D coordinates of the central point for the red marker in R1; ${\bar{x}}_{r_{-}C}$ and ${\bar{y}}_{r_{-}C}$ are the 2D coordinates of the central point for the cyan marker in R1. The numbers of corners in $S_{l_{-}\mathrm{RC}}$ and $S_{r_{-}\mathrm{RC}}$ can be denoted as $N_{l\_corner\_RC}$ and $N_{r\_corner\_RC}$, wherein $1<N_{l\_corner\_RC}<N_{l\_corner}$, $1<N_{r\_corner\_RC}<N_{r\_corner}$ and $N_{l\_corner\_RC}=N_{r\_corner\_RC}$.

The corner sets $S_{l_{-}RC}$ and $S_{r_{-}RC}$ in the left- and right-view images for the same baseline are acquired through regional constraint, wherein $S_{l_{-}RC}\subset S_{l}$ and $S_{r_{-}RC}\subset S_{r}$. According to the characteristic of the checkerboard, the x coordinates of the corners on the same line increase successively. Therefore, the corners in the corner sets $S_{l_{-}RC}$ and $S_{r_{-}RC}$ are ordered by the x coordinate, as expressed in Equations (9) and (10); and the pixels of the same corner in the left- and right-view images correspond in order. That is, the ordered $z_{l\_\mathrm{corner}\_\mathrm{RC}\_i}$ and $z_{r\_\mathrm{corner}\_\mathrm{RC}\_i}$ with the same i correspond to the same corner in 3D space, and they are a stereo-matching point pair. Thus, refined stereo matching at the sub-pixel level can be achieved, and with less complexity. Algorithm 1 describes the refined stereo-matching process described above.

(9) $$\begin{array}{c} z_{l\_\mathrm{corner}\_\mathrm{RC}\_1},\ldots ,z_{l\_\mathrm{corner}\_\mathrm{RC}\_i},\ldots ,z_{l\_\mathrm{corner}\_\mathrm{RC}\_N_{l\_\mathrm{corner}\_\mathrm{RC}}}\;\;\;\;\;\;\;\;\;\;\;\;\\ s.t.\;\;x_{l\_corner\_RC\_1}<\ldots <x_{l\_corner\_RC\_i}<\ldots <x_{l\_corner\_RC\_N_{l\_corner\_RC}} \end{array}$$

(10) $$\begin{array}{c} z_{r\_\mathrm{corner}\_\mathrm{RC}\_1},\ldots ,z_{r\_\mathrm{corner}\_\mathrm{RC}\_i},\ldots ,z_{r\_\mathrm{corner}\_\mathrm{RC}\_N_{r\_\mathrm{corner}\_\mathrm{RC}}}\;\;\;\;\;\;\;\;\;\;\;\;\\ s.t.\;\;x_{r\_corner\_RC\_1}<\ldots <x_{r\_corner\_RC\_i}<\ldots <x_{r\_corner\_RC\_N_{r\_corner\_RC}} \end{array}$$

To further increase the anthropometry accuracy, multiple measurements can be carried out on the same girth so that the optimal value can be selected from multiple measurement results. Hence, it is necessary to match multiple lines of corners precisely and simply. The central points of the red and cyan markers are moved up or down along the y direction in a step $N_{\mathrm{step}\;}$, wherein $N_{\mathrm{step}\;}$ is the pixel difference corresponding to the checkerboard interval in the image. $N_{\mathrm{step}\;}$ is inversely proportional to the shooting distance D(m), and the relationship is shown in Equation (11):

(11) $$N_{\mathrm{step}\;}=7.02D^{2}-45.18D+93.43$$

In the experiment, D=2.4 m and $N_{\mathrm{step}}=25$ pixels. The y coordinates of ${\bar{z}}_{l_{-}R}\left({\bar{x}}_{l_{-}R},{\bar{y}}_{l_{-}R}\right)$, ${\bar{z}}_{r_{-}R}\left({\bar{x}}_{r_{-}R},{\bar{y}}_{r_{-}R}\right)$, ${\bar{z}}_{l_{-}C}\left({\bar{x}}_{l_{-}C},{\bar{y}}_{l_{-}C}\right)$ and ${\bar{z}}_{r_{-}C}\left({\bar{x}}_{r_{-}C},{\bar{y}}_{r_{-}C}\right)$ in the left- and right-view images increased or decrease upward or downward in the step $N_{\mathrm{step}\;}$ to get ${\bar{z}}_{l_{-}R}{}^{\prime }\left({\bar{x}}_{l_{-}R},{\bar{y}}_{l_{-}R}\pm N_{\mathrm{step}\;}\right)$, ${\bar{z}}_{r_{-}R}{}^{\prime }\left({\bar{x}}_{r_{-}R},{\bar{y}}_{r_{-}R}\pm N_{\mathrm{step}\;}\right)$, ${\bar{z}}_{l_{-}C}{}^{\prime }\left({\bar{x}}_{l_{-}C},{\bar{y}}_{l_{-}C}\pm N_{\mathrm{step}\;}\right)$ and ${\bar{z}}_{r_{-}C}{}^{\prime }\left({\bar{x}}_{r_{-}C},{\bar{y}}_{r_{-}C}\pm N_{\mathrm{step}\;}\right)$. Then, accurate matching of the other two lines of corners in the same segmented region was achieved in the way described above.

By using the binocular calibration parameters, the 3D coordinates of each line of stereo-matching corner pairs were calculated; then the corners were reversely rotated back to the initial positions according to the rotation angle of the turntable. Next, the PIVCF curve fitting method was used to achieve human-body girth fitting, and finally, the human-body parameter measurement data of multiple lines in the same region were calculated. According to GB/T 16160-2017 [48] “Anthropometric Definitions and Methods for Garment”, the maximum girth data among the three is output as the final girth measurements of bust, hip and thigh, and the minimum girth data are output as the final girth measurement of the waist. Moreover, by moving down the measure line of bust or thigh in 2$N_{\mathrm{step}\;}$, the girth data of the third line are output as the final girth measurements of under-bust or mid-thigh, respectively.
**Algorithm 1** The refined stereo-matching process.**Input:** Segmented images L1 and R1; **Output:** Stereo-matching point pairs;  1:Extract pixel sets of the red and cyan markers according to *H*, *S*, *V* components, $M_{l_{-}R}$ and $M_{l_{-}C}$ for L1, $M_{r_{-}R}$ and $M_{r_{-}C}$ for R1;  2:Calculate the central points ${\bar{z}}_{l_{-}R}$, ${\bar{z}}_{r_{-}R}$, ${\bar{z}}_{l_{-}C}$ and ${\bar{z}}_{r_{-}C}$ for $M_{l_{-}R}$, $M_{r_{-}R}$, $M_{l_{-}C}$ and $M_{r_{-}C}$, respectively;  3:Extract corner sets $S_{l}$ and $S_{r}$ for L1 and R1 by Shi–Tomasi corner detection algorithm;  4:Get a smaller corner set $S_{l_{-}RC}$ constrained by ${\bar{z}}_{l_{-}R}$ and ${\bar{z}}_{l_{-}C}$ from $S_{l}$, and another smaller corner set $S_{r_{-}RC}$ constrained by ${\bar{z}}_{r_{-}R}$ and ${\bar{z}}_{r_{-}C}$ from $S_{r}$;  5:Order the corners in $S_{l_{-}RC}$ and $S_{r_{-}RC}$ separately according to the x coordinates of the corners;  6:**return** The stereo-matching point pairs $\left(z_{l\_\mathrm{corner}\_\mathrm{RC}\_1},z_{r\_\mathrm{corner}\_\mathrm{RC}\_1}\right),$$\ldots ,\left(z_{l\_\mathrm{corner}\_\mathrm{RC}\_i},z_{r\_\mathrm{corner}\_\mathrm{RC}\_i}\right),\ldots ,\left(z_{l\_\mathrm{corner}\_\mathrm{RC}\_N_{l\_\mathrm{corner}\_\mathrm{RC}}},z_{r\_\mathrm{corner}\_\mathrm{RC}\_N_{r\_\mathrm{corner}\_\mathrm{RC}}}\right)$.

## 4. Experiments

In the practical girth measurement experiment, the size manually measured in accordance with GB/T 16160-2017 was chosen as the ground truth. The practical girth measurement system consisted of two Hikvision MV-CA050-11UC industrial cameras; a precise revolving platform; and a laptop with an Intel(R) Core (TM) i7-10750H CPU, a 16G RAM and a NVIDIA GeForce RTX 2060 discrete graphics card. We used a NVIDIA 2080Ti GPU and an Intel E5 2678 V3 CPU for training and testing. Our model was implemented on Pytorch with Python3 under Windows10. We utilized Zhengyou Zhang’s calibration method [49] to calibrate the binocular stereovision camera by means of a calibration board with a cell size of 30 mm. To avoid random errors, each subject was measured manually and by our system five times each, and the average value was calculated as the final measurement data. The mean absolute difference (MAD) [50] was used to measure the difference between the measurement data and the ground truth. Forty-eight young subjects aged from 20 to 30 years old without obvious physical abnormalities were randomly selected; 25 were males and 23 were females. Six girths were measured for each subject, including bust, under-bust, waist, hip, thigh and mid-thigh. The girth measurements were divided into two groups: male and female. For simplicity, only 10 measurement results are shown below, including those with the maximum absolute errors.

### 4.1. Girth Measurement Experiment for Males

Table 4 shows the girth measurement results of 10 subjects selected from the 25 males, including six subjects with the maximum absolute error of bust, under-bust, waist, hip, thigh, and mid-thigh measurements. The remaining four subjects were randomly selected. Male subject 4 had the maximum absolute error of bust, i.e., 1.43 cm, which conforms to China’s national standard GB/T 2664-2017, “Men’s suits and coats”, a ±2.0 cm tolerance for the bust [51]. Male subject 8 had the maximum absolute error of the under-bust, i.e., 1.59 cm, which conforms to China’s textile industry standard FZ/T 73017-2014, “Knitted homewear”, a ±2.0 cm tolerance for a width above 5 cm [52]. Male subject 3 had the maximum absolute error of waist, i.e., 1.49 cm, which conforms to China’s textile industry standard FZ/T 73029-2019, “Knitted leggings”, a ±2.0 cm tolerance for the waist [53]. Male subject 2 had the maximum absolute error of hip, i.e., 1.50 cm, which conforms to China textile industry standard FZ/T 73022-2019, “Knitted thermal underwear”, a ±2.0 cm tolerance for the hip [54]. Male subject 1 had the maximum absolute error of the thigh, i.e., 1.47 cm, and male subject 9 had the maximum absolute error of the mid-thigh, i.e., 1.15 cm, which also conform to FZ/T 73017-2014—the ±2.0 cm tolerance for the width above 5 cm.

**Figure 14:**
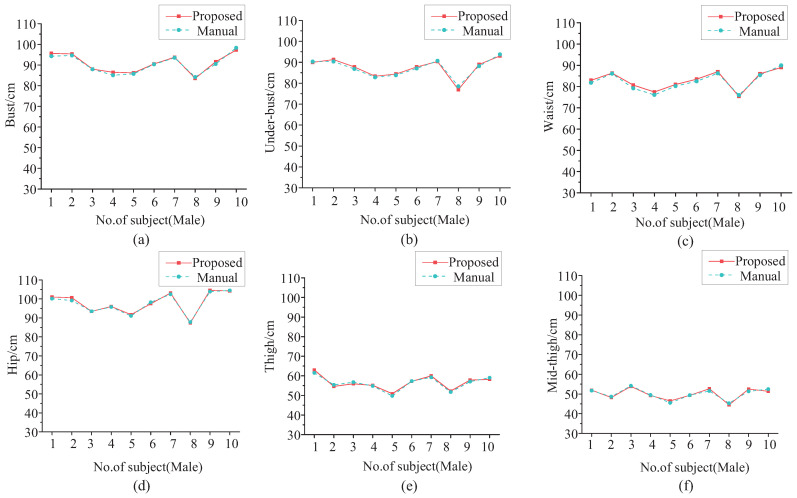
Girth measurement results comparison for males. (**a**) Bust. (**b**) Under-bust. (**c**) Waist. (**d**) Hip. (**e**) Thigh. (**f**) Mid-thigh.

Figure 14 shows the comparison of the six girth measurement results of these 10 male subjects for our proposed method and the manual method. The red line with squares represents the measurement results by the proposed method, and the cyan dotted line with circles represents the manual measurement results. The two lines are very close and almost overlapping. Table 5 shows the statistical analysis of the girth measurement results of the 25 male subjects. The mean values (μ) and standard deviations (σ) of the measurement results are almost the same for the proposed method and the manual method, which indicates that the proposed method can replace the manual method.

### 4.2. Girth Measurement Experiment for Females

Table 6 shows the girth measurement results of 10 subjects selected from the 23 female subjects, including four subjects with the maximum absolute error of the bust, under-bust, waist, hip, thigh and mid-thigh. The remaining six subjects were randomly selected. Female subject 15 had the maximum absolute error of bust, i.e., 1.42 cm, which conforms to China national standard GB/T 2665-2017, “Women’s suits and coats”—a ±2.0 cm tolerance for bust [55]. Female subject 14 had the maximum absolute error of the under-bust, i.e., 1.47 cm, which conforms to FZ/T 73017-2014—a ±2.0 cm tolerance for a width above 5 cm [52]. Female subject 16 had the maximum absolute error of waist, i.e., 1.34 cm, which conforms to FZ/T 73029-2019—a ±2.0 cm tolerance for the waist [53]. Female subject 14 also had the maximum absolute error of the hip, i.e., 1.30cm, which conformed to FZ/T 73022-2019—a ±2.0 cm tolerance for the hip [54]. Female subject 18 had the maximum absolute error of the thigh, i.e., 1.34cm, and female subject 18 had the maximum absolute error of the mid-thigh, i.e., 0.71 cm, which also conform to FZ/T 73017-2014—the ±2.0 cm tolerance for the width above 5 cm.

**Figure 15:**
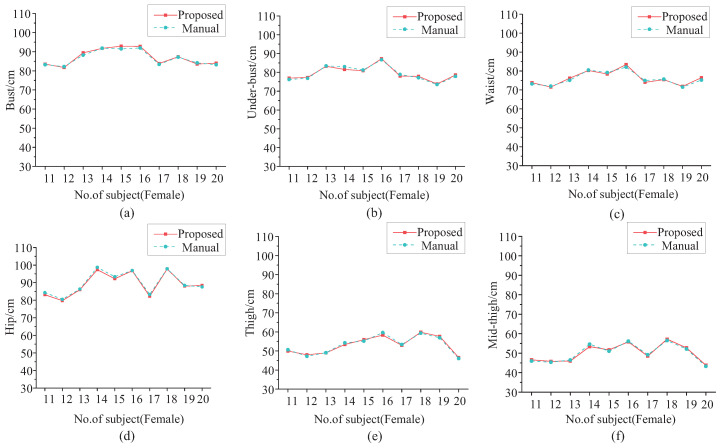
Girth measurement results comparison for females. (**a**) Bust. (**b**) Under-bust. (**c**) Waist. (**d**) Hip. (**e**) Thigh. (**f**) Mid-thigh.

Figure 15 shows the comparison of the six girth measurement results of these 10 female subjects for our proposed method and the manual method. The red line with squares represents the measurement results by the proposed method, and the cyan dotted line with circles represents the manual measurement results. The two lines are very close and almost overlapping. Table 7 shows the statistical analysis of the girth measurement results of the 23 female subjects. The mean values (μ) and standard deviations (σ) of the measurement results are almost the same for the proposed method and the manual method, which indicates that the proposed method can replace the manual method.

In conclusion, the maximum measurement error of the bust was 1.43 cm for males and 1.42 cm for females, which are within the ±2.0 cm tolerance for the bust for males and females regulated by the national standards. The maximum measurement error of under-bust was 1.59 cm for males and 1.47cm for females, which are within the ±2.0 cm tolerance for the under-bust regulated by the textile industry standard. The maximum measurement error of the waist was 1.49 cm for males and 1.34 cm for females, which are within the ±2.0 cm tolerance for the waist regulated by the textile industry standard. The maximum measurement error of hip was 1.5 cm for males and 1.30 cm for females, which are within the ±2.0 cm tolerance for the hip regulated by the textile industry standard. The maximum measurement error of the thigh was 1.47 cm for males and 1.34 cm for females, which are within the ±2.0 cm tolerance for the thigh regulated by the textile industry standard. The maximum measurement error of the mid-thigh was 1.15 cm for males and 0.71 cm for females, which are within the ±2.0 cm tolerance for the thigh regulated by the textile industry standard.

As shown in Table 8, the girth measurement errors of the bust, waist and hip when using the proposed method and five other anthropometric methods are compared, namely, Han et al.’s method [56], Lu et al.’s method [57], Kaashki et al.’s method [58], Yang et al.’s method [17] and Song et al.’s method [18]. The bust MAD of our system was 0.66 cm, which is less than the bust MAD values of [17,18,56,57,58], which were 0.99, 1.60, 1.97, 1.11 and 1.45 cm, respectively. The waist MAD of our improved system was 0.76cm, which is less than the waist MAD values of [17,18,56,57,58], which were 0.85, 1.20, 2.03, 1.03 and 1.47 cm, respectively. The hip MAD of our improved system was 0.68 cm, which is less than the hip MAD values of [18,56,57,58], which were 1.15, 1.12, 0.91 and 1.02 cm, respectively. In summary, our system improves the anthropometric system by improving the human-body-segmentation algorithm with attention-based feature fusion and by refining the stereo-matching scheme to the sub-pixel level. Not only can our system measure the girth simply and intelligently with low cost and portability, but it also can achieve better measurement accuracy than other methods.

## 5. Conclusions

In this study, to further increase the anthropometric accuracy, we improved the semantic segmentation process in the anthropometric system by a human-body-segmentation algorithm with attention-based feature fusion and improved the stereo matching and coordinate calculation process through a refined corner-based feature-point design with sub-pixel stereo matching. We proposed a CBAM-PSPNet which could increase the accuracy and decrease the computational cost of the human-body-segmentation algorithm PSPNet. We designed a refined stereo-matching scheme based on the corner feature point which could enhance the accuracy and reduce the complexity of the stereo-matching method. The girth measurement performance of our proposed system was verified by the experiments measuring the bust, under-bust, waist, hip, thigh and mid-thigh on males and females. The results show that our system is efficient and reliable. In our measurements, the measured girths all had a maximum girth absolute error within the ±2.0 cm error limit of the corresponding national standard or textile industry standard. The girth measurement errors are also smaller than those of other methods. In particular, our proposed CBAM-PSPNet and corner-based stereo-matching method effectively improve the accuracy and efficiency of the anthropometric system.

## Figures and Tables

**Table 1:** Number of parameters and computational cost for the improved ResNet101 and the ResNet101.

Backbone	Number of Parameters (Million)	FLOPs (Billion)
ResNet101	42.50	7.84
Improved ResNet101	32.52	5.94

**Table 2:** Performance comparison between CBAM-PSPNet and PSPNet.

Network	PA (%)	MPA (%)	MIOU (%)
PSPNet	98.36	88.25	82.30
CBAM-PSPNet	98.39	92.28	83.11

**Table 3:** HSV ranges corresponding to the four colors.

	$H_{\min}$	$H_{\max}$	$S_{\min}$	$S_{\max}$	$V_{\min}$	$V_{\max}$
Red	0/156	10/180	45	255	46	255
Cyan	78	99	43	255	46	255
Black	0	180	0	255	0	46
White	0	180	0	30	221	225

**Table 4:** The exemplary girth measurement results of 10 male subjects.

**NO.**	**Girth**	**Proposed (cm)**	**Manual (cm**)	**Error (cm)**	**Error Rate (%)**
1	bust	95.64	94.3	−1.34	−1.42
	under-bust	89.94	90.3	0.36	0.40
	waist	82.95	81.8	−1.15	−1.41
	hip	101	100.14	−0.86	−0.86
	thigh	62.97	61.5	−1.47	−2.39
	mid-thigh	51.93	51.7	−0.23	−0.44
2	bust	95.37	94.66	−0.71	−0.75
	under-bust	91.28	90.36	−0.92	−1.02
	waist	86.29	86.08	−0.21	−0.24
	hip	100.66	99.16	−1.5	−1.51
	thigh	54.61	55.37	0.76	1.37
	mid-thigh	48.21	48.62	0.41	0.84
3	bust	88.02	87.97	−0.05	−0.06
	under-bust	87.76	86.8	−0.96	−1.11
	waist	80.69	79.2	−1.49	−1.88
	hip	93.43	93.5	0.07	0.07
	thigh	55.91	56.76	0.85	1.50
	mid-thigh	53.86	54.18	0.32	0.59
4	bust	86.49	85.06	−1.43	−1.68
	under-bust	83.33	82.8	−0.53	−0.64
	waist	77.45	76.1	−1.35	−1.77
	hip	96.02	95.73	−0.29	−0.30
	thigh	55.13	54.8	−0.33	−0.60
	mid-thigh	49.23	49.5	0.27	0.55
**NO.**	**Girth**	**Proposed (cm)**	**Manual (cm**)	**Error (cm)**	**Error rate (%)**
5	bust	86.21	85.74	−0.47	−0.55
	under-bust	84.36	83.82	−0.54	−0.64
	waist	80.99	80.2	−0.79	−0.99
	hip	91.72	91.02	−0.7	−0.77
	thigh	50.88	49.74	−1.14	−2.29
	mid-thigh	46.51	45.5	−1.01	−2.22
6	bust	90.53	90.34	−0.19	−0.21
	under-bust	87.75	87.04	−0.71	−0.82
	waist	83.46	82.5	−0.96	−1.16
	hip	97.47	98.25	0.78	0.79
	thigh	57.16	57.3	0.14	0.24
	mid-thigh	49.3	49.38	0.08	0.16
7	bust	93.76	93.5	−0.26	−0.28
	under-bust	90.32	90.70	0.38	0.42
	waist	87.01	86.22	−0.79	−0.92
	hip	103.1	102.43	−0.67	−0.65
	thigh	59.97	59.25	−0.72	−1.22
	mid-thigh	52.66	51.52	−1.14	−2.21
8	bust	83.56	84.14	0.58	0.69
	under-bust	76.93	78.52	1.59	2.02
	waist	75.38	75.98	0.6	0.79
	hip	87.37	87.8	0.43	0.49
	thigh	52.22	51.7	−0.52	−1.01
	mid-thigh	44.52	45.30	0.78	1.72
9	bust	91.55	90.48	−1.07	−1.18
	under-bust	88.96	88.16	−0.80	−0.91
	waist	86.02	85.36	−0.66	−0.77
	hip	104.55	103.84	−0.71	−0.68
	thigh	57.83	57.05	−0.78	−1.37
	mid-thigh	52.49	51.34	−1.15	−2.24
10	bust	97.28	98.32	1.04	1.06
	under-bust	92.99	93.74	0.75	0.80
	waist	88.96	89.94	0.98	1.09
	hip	104.2	104.48	0.28	0.27
	thigh	58.21	59.02	0.81	1.37
	mid-thigh	51.37	52.46	1.09	2.08

**Table 5:** Statistical analysis of the girth measurement results of 25 male subjects.

Male				
		μ **(cm)**	σ **(cm)**	**MAD (cm)**
Bust	proposed	90.84	4.42	0.71
	manual	90.45	4.48	
Under-bust	proposed	87.36	4.47	0.75
	manual	87.22	4.26	
Waist	proposed	82.92	4.12	0.90
	manual	82.34	4.36	
Hip	proposed	97.95	5.49	0.63
	manual	97.64	5.28	
Thigh	proposed	56.49	3.39	0.75
	manual	56.25	3.35	
Mid-thigh	proposed	50.00	2.83	0.65
	manual	49.95	2.75	

**Table 6:** The exemplary girth measurement results of 10 female subjects.

**NO.**	**Girth**	**Proposed (cm)**	**Manual (cm)**	**Error (cm)**	**Error Rate (%)**
11	bust	83.51	83.24	0.27	0.32
	under-bust	76.94	76.22	0.72	0.94
	waist	73.83	73.16	0.67	0.92
	hip	83.1	84.32	−1.22	−1.45
	thigh	49.94	50.63	−0.69	−1.36
	mid-thigh	46.58	45.96	0.62	1.35
12	bust	81.77	82.2	−0.43	−0.52
	under-bust	77.26	76.84	0.42	0.55
	waist	71.52	72.02	−0.5	−0.69
	hip	79.75	80.5	−0.75	−0.93
	thigh	47.99	47.2	0.79	1.67
	mid-thigh	45.83	45.36	0.47	1.04
13	bust	89.43	88.25	1.18	1.34
	under-bust	83.22	83.42	−0.2	−0.24
	waist	76.2	75.12	1.08	1.44
	hip	85.96	86.34	−0.38	−0.44
	thigh	48.98	49.06	−0.08	−0.16
	mid-thigh	45.98	46.5	−0.52	−1.12
14	bust	91.77	91.84	−0.07	−0.08
	under-bust	81.55	83.02	−1.47	−1.77
	waist	80.27	80.5	−0.23	−0.29
	hip	97.4	98.7	−1.3	−1.32
	thigh	53.38	54.62	−1.24	−2.27
	mid-thigh	52.33	52.08	0.25	0.48
15	bust	92.92	91.5	1.42	1.55
	under-bust	80.86	81.25	−0.39	−0.48
	waist	78.39	79.18	−0.79	−1.00
	hip	92.21	93.38	−1.17	−1.25
	thigh	55.9	55.18	0.72	1.30
	mid-thigh	51.69	51.02	0.67	1.31
**NO.**	**Girth**	**Proposed (cm)**	**Manual (cm)**	**Error (cm)**	**Error Rate (%)**
16	bust	92.82	91.96	0.86	0.94
	under_bust	87.24	86.68	0.56	0.65
	waist	83.4	82.06	1.34	1.63
	hip	96.78	96.92	−0.14	−0.14
	thigh	58.3	59.64	−1.34	−2.25
	mid-thigh	55.82	56.16	−0.34	−0.61
17	bust	83.86	83.42	0.44	0.53
	under-bust	78.06	78.9	−0.84	−1.06
	waist	74.01	74.98	−0.97	−1.29
	hip	82.22	83.28	−1.06	−1.27
	thigh	52.96	53.36	−0.4	−0.75
	mid-thigh	48.51	49.2	−0.69	−1.40
18	bust	87.36	87.18	0.18	0.21
	under-bust	77.85	77.12	0.73	0.95
	waist	75.44	75.7	−0.26	−0.34
	hip	97.8	97.9	−0.1	−0.10
	thigh	59.81	59.32	0.49	0.83
	mid-thigh	57.21	56.5	0.71	1.26
19	bust	83.56	84.16	−0.6	−0.71
	under-bust	73.82	73.54	0.28	0.38
	waist	71.89	71.5	0.39	0.55
	hip	87.99	88.42	−0.43	−0.49
	thigh	57.65	56.92	0.73	1.28
	mid-thigh	52.73	52.16	0.57	1.09
20	bust	83.98	83.22	0.76	0.91
	under-bust	78.61	77.87	0.74	0.95
	waist	76.5	75.24	1.26	1.67
	hip	88.43	87.6	0.83	0.95
	thigh	46.53	46.04	0.49	1.06
	mid-thigh	43.81	43.26	0.55	1.27

**Table 7:** Statistical analysis of the girth measurement results of 23 female subjects.

Female				
		μ **(cm)**	σ **(cm)**	**MAD (cm)**
Bust	proposed	87.10	4.10	0.61
	manual	86.70	3.76	
Under-bust	proposed	79.35	3.59	0.66
	manual	79.28	3.80	
Waist	proposed	76.15	3.53	0.64
	manual	75.95	3.37	
Hip	proposed	89.16	6.29	0.74
	manual	89.74	6.21	
Thigh	proposed	53.14	4.44	0.66
	manual	53.12	4.57	
Mid-thigh	proposed	50.05	4.39	0.64
	manual	49.82	4.51	

**Table 8:** Error comparison for girth measurement.

	MAD (cm)		
	Bust	Waist	Hip
Han et al. [56]	1.97	2.03	1.12
Lu et al. [57]	1.11	1.03	0.91
Kaashki et al. [58]	1.45	1.47	1.02
Yang et al. [17]	0.99	0.85	NA
Song et al. [18]	1.60	1.20	1.15
Proposed system	0.66	0.76	0.68

## Data Availability

Not applicable.
